# A case of mitomycin C toxicity after XEN gel stent implantation with the XEN air technique in a glaucoma patient

**DOI:** 10.1186/s12886-023-03152-4

**Published:** 2023-10-10

**Authors:** Joon Young Baeg, Han Sol Choi, Si Hyung Lee

**Affiliations:** 1 Department of Ophthalmology, College of Medicine, Soonchunhyang University, Cheonan, Republic of Korea; 2 Department of Ophthalmology, Soonchunhyang University Hospital Bucheon, Bucheon, Republic of Korea

**Keywords:** Postsurgical complication, Uveitic glaucoma, XEN gel stent

## Abstract

**Background:**

To discuss the first case of mitomycin C (MMC) toxicity after XEN® gel stent implantation in a glaucoma patient, conducted using the XEN “air” technique with an ophthalmic viscosurgical device (OVD).

**Case presentation:**

A 44-year-old Asian male presented with increased intraocular pressure (IOP; 52 mmHg) accompanied by keratic precipitates and an edematous cornea. He was diagnosed with uveitic glaucoma in the left eye, and the IOP was controlled with a topical anti-glaucoma agent. However, glaucoma progression was revealed by Humphrey visual field (HVF) and optical coherence tomography (OCT) examinations. The patient underwent uneventful XEN gel stent implantation using the XEN air technique and an MMC (0.02%, 0.1 mL) injection, with subconjunctival air and OVD injection provided prior to XEN implantation in the left eye. The patient exhibited a decreased IOP (11 mmHg), elevated bleb, and extensive subconjunctival hemorrhage on postoperative day 1. On postoperative day 18, diffuse conjunctival injection and a large avascular bleb was noticed around the XEN gel stent. The patient complained of severe eye pain and discomfort, suggestive of MMC toxicity, and the IOP was 12 mmHg. The patient was treated with a topical steroid and antibiotics tapered over a 6-month period. Finally, the toxicity was successfully controlled, with the IOP stabilizing at around 15 mmHg.

**Conclusions:**

Although significantly greater lowering of the IOP can be expected with the use of subconjunctival OVD injection and MMC during XEN gel stent implantation, a cautious approach and a longer monitoring period are required.

## Background

Glaucoma is a major vision-threatening optic neuropathy that causes irreversible vision loss. Topical anti-glaucoma agents are commonly considered the first line treatment; however, uncontrolled intraocular pressure (IOP) usually necessitates laser treatment and surgical management, including trabeculectomy and Ahmed glaucoma valve implantation. Minimally invasive glaucoma surgery (MIGS), which was introduced recently but is already widely used, has demonstrated efficacy for lowering the IOP of glaucoma patients. The XEN® gel stent (Allergan Inc., Dublin, Ireland) MIGS device is used worldwide. Recently, Vera et al. introduced the XEN “air” technique, which was designed to maintain long-term bleb stability through pneumatic and viscoelastic dissection of the conjunctiva and Tenon’s capsule prior to XEN gel stent implantation [1]. During surgery, mitomycin C (MMC) is used to reduce post-operative subconjunctival fibrosis and scar formation in the filtering bleb, which is an essential aspect of glaucoma filtration surgery. However, the “anti-metabolite” filtration surgery may result in major complications and side effects, such as thin-walled cystic blebs, late bleb leaks, bleb infections, endophthalmitis, chronic hypotony, and persistent corneal epithelial defects [2–4]. Here, we present a case of MMC toxicity that occurred after XEN gel stent implantation using the XEN air technique, presumably due to ophthalmic viscosurgical device (OVD) usage.

## Case presentation

A 44-year-old Asian male patient was referred to the outpatient department with an elevated IOP (52 mmHg) in the left eye that had occurred 3 days prior. He had a history of uveitis in his left eye 2 years ago. The best-corrected visual acuity was 20/20 and 20/25 in the right and left eyes, respectively. Keratic precipitates (KP) were accompanied by conjunctival injection and anterior chamber cell reaction in the left eye. Fundus examination showed superotemporal and inferotemporal retinal nerve fiber layer (RNFL) defects in the left eye, as confirmed by optical coherence tomography (OCT, Topcon Medical Systems Inc., Oakland, NJ, USA) examination. On the Humphrey visual field (HVF, Carl Zeiss Meditec Inc., Dublin, USA) test, a visual field defect was found; inferior nasal field scotoma extended to the arcuate scotoma in the left eye. The patient was diagnosed with uveitic glaucoma in the left eye. Topical anti-glaucomatic treatment using tafluprost/timolol fixed combination (Tapcom, Santen Pharm. Co., Ltd., Osaka, Japan), brimonidine/brinzolamide fixed combination (Simbrinza, Novatis Co., Basel, Swiss) and topical treatment using prednisolone acetate 1%(Predbell, Chong Kun Dang Pharm. Co., Seoul, Korea) eyedrops were initiated, with close observation of glaucoma progression at the follow-up visits. Serial examinations, including blood test, OCT, and HVF test, were conducted. All factors including HLA-B27 were negative in the blood test. During the 2-year follow-up period, the IOP in the left eye intermittently increased to 60 mmHg, and enlargement of the scotoma and RNFL defect were noticed on HVF and OCT examinations, respectively, indicative of glaucoma progression. There was no evidence of cataract progression during the entire follow-up period which could affect the result of HVF test. Surgical intervention was considered to control glaucoma.

**Fig. 1:**
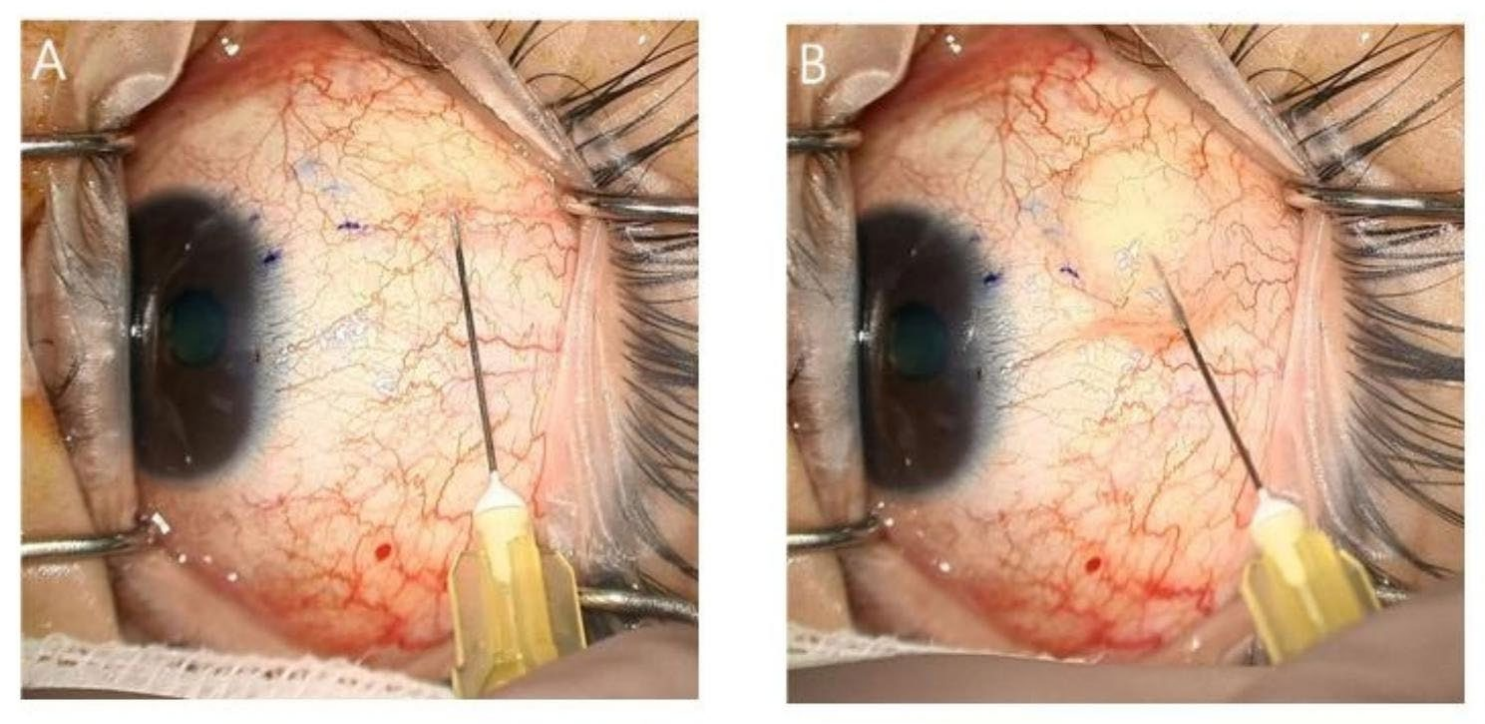
XEN “air” technique. Subconjunctival air (**A**) and the ophthalmic viscosurgical device are shown. (**B**) Injection at the superonasal quadrant prior to mitomycin **C** (MMC) injection

The patient requested surgical management for prompt recovery. Since patient wanted to minimize any incisional process during surgical procedure, XEN gel stent implantation was considered in conjunction with subconjunctival MMC and OVD injection. Regarding the intraoperative approach, gentian violet staining of the conjunctiva at 3.0 mm away from the limbus was performed first. A 30-gauge needle was superficially placed under the conjunctiva, which was connected to an air/OVD-filled syringe. Slowly, air was injected into the subconjunctival space for pneumatic dissection of the conjunctiva and Tenon’s capsule. Afterwards, the OVD was placed in the space created by the air, thus making a subconjunctival pocket for the XEN gel stent to be placed just above Tenon’s capsule (Fig. 1). The injector needle was introduced into the anterior chamber and penetrated slightly anterior to the scleral spur, assisted by a gonioscopy lens. An approximately 1-mm length of the XEN gel stent was left in the anterior chamber, and the remnant was inserted at the superonasal quadrant of the subconjunctival area; a good stent position was confirmed. Diluted MMC (0.02%, 0.1 mL) was injected into the subconjunctival space where the prior air/OVD was injected. The patient was started on gatifloxacin (0.3% Gatiflo, Samil Pharm. Co., Ltd., Seoul, Korea) and loteprednol (0.5% Lotemax, Bausch & Lomb, Bridgewater, NJ, USA) eyedrops four times daily; the anti-glaucoma agent was stopped.

**Fig. 2:**
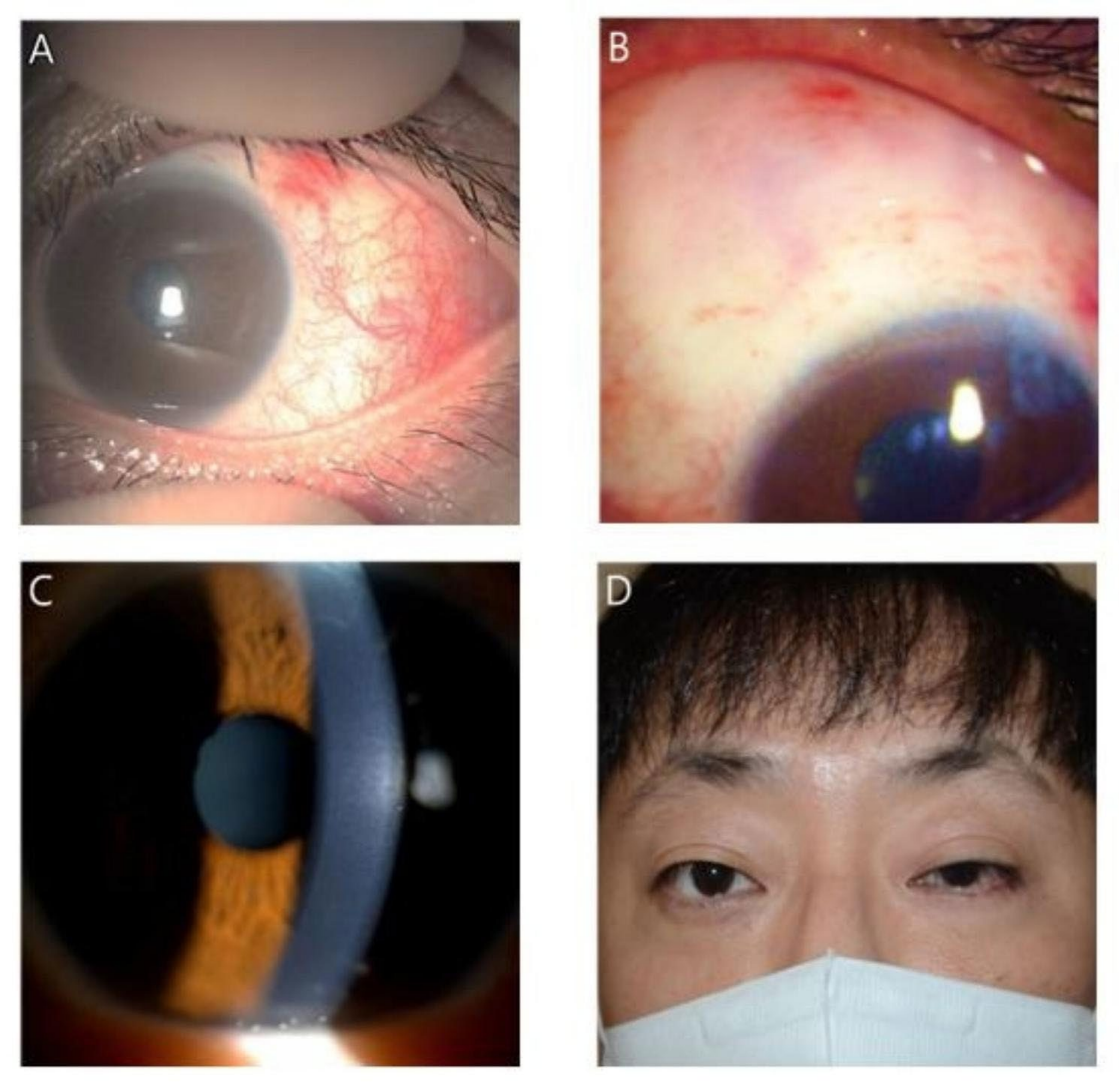
Signs of MMC toxicity included diffuse conjunctival injection (**A**), a large avascular bleb (**B**), mild corneal stromal haze (**C**), and persistent upper eyelid ptosis (**D**) at 3 months after the surgery

**Fig. 3:**
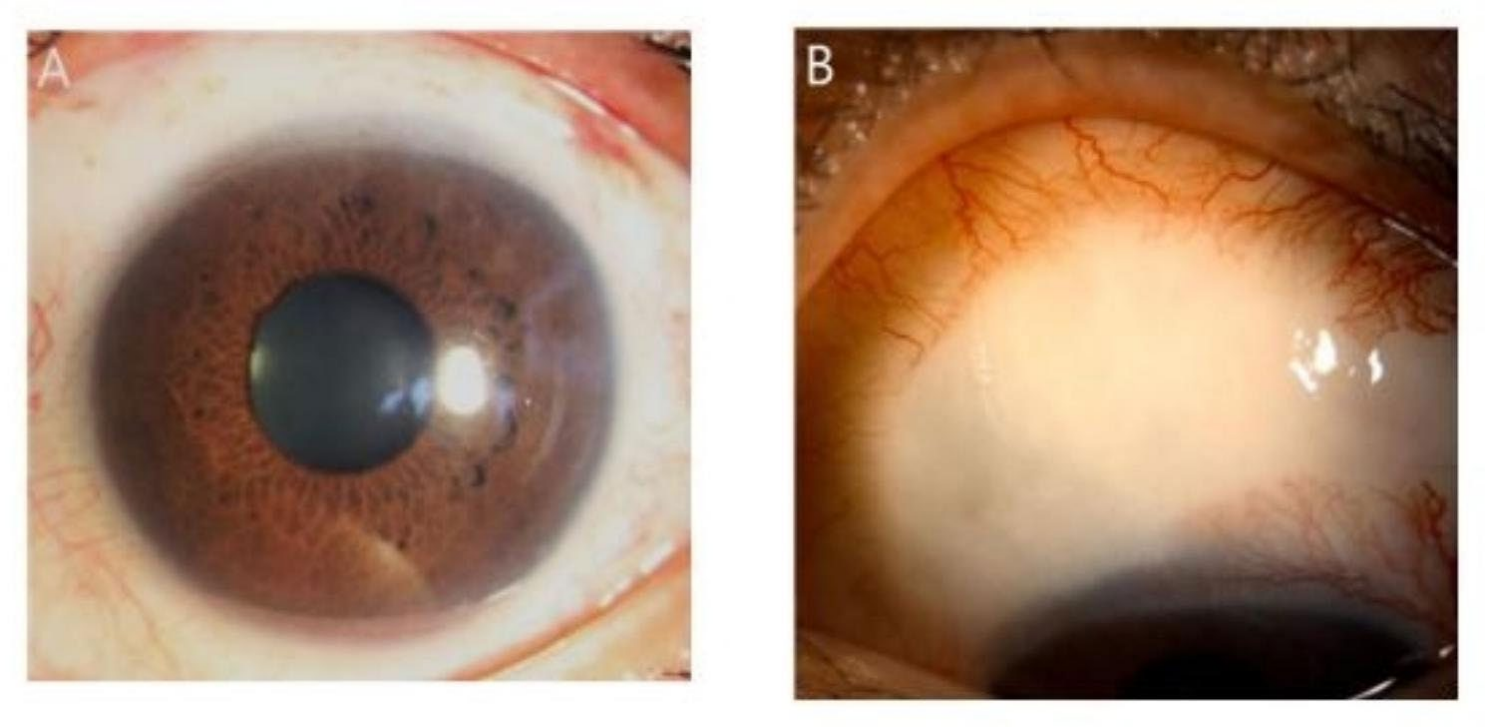
Improved subconjunctival injection (**A**) and a large avascular bleb (**B**) at 6 months after the surgery

One day after surgery, the IOP was controlled to 11 mmHg with mild conjunctival injection and subconjunctival hemorrhage. The bleb appeared well-elevated with underneath XEN gel stent visible. However, on postoperative day 18, diffuse conjunctival hyperemia and an avascular bleb was noticed, and the patient reported photophobia and ocular pain. We considered these signs and symptoms as MMC toxicity but not as those of recurrence of uveitis since the patient did not present any signs of anterior chamber reaction, hypopyon, infiltration or KPs from the early postoperative period, which are common signs of uveitis. Topical loteprednol was exchanged with prednisolone (1%, Predbell, Chong Kun Dang Pharm. Co., Seoul, Korea) eyedrop every 2 h to control the suspected MMC toxicity. The patient underwent close observation at weekly intervals. The left eye showed slight improvement in terms of conjunctival hyperemia at 1 month postoperatively. The IOP of the left eye was well controlled in the range of 16–18 mmHg, and prednisolone eyedrop instillation was adjusted to four times a day. However, diffuse conjunctival hyperemia persisted for several months, with surrounding injection around avascular bleb, mild corneal stromal haze, and the patient complained of continuous eyeball pain (Fig. 2A-C). Additionally, a mild to moderate degree of ptosis occurred on the left upper eyelid after 3 months (Fig. 2D). During this period, there was no signs of an infection or recurrence of uveitis, such as keratic precipitates, anterior chamber flare, hypopyon, infiltration, or discharge. The inflammation subsided only after 6 months, with the left eye showing a clear cornea and avascular bleb; the patient did not complain of any ocular discomfort. The IOP at 6 months after surgery was 15 mmHg. Topical anti-inflammatory eyedrops were changed to levofloxacin (1.5% Cravit, Santen Pharm. Co., Ltd., Osaka, Japan), loteprednol, and BAK-free brimonidine (0.15%; Bridin-T, Hanlim Pharm. Co., Ltd., Seoul, Korea) was added. The patient is still attending regular follow-ups to check for other possible complications (Fig. 3).

## Discussion and conclusions

The XEN gel stent is a 45-µm-diameter, 6-mm-long hydrophilic stent consisting of cross-linked porcine gelatin [5]. It has rarely been associated with early postoperative complications such as a shallow anterior chamber, wound leak, aqueous misdirection, or suprachoroidal/vitreous hemorrhage, which are commonly seen with tube surgery or trabeculectomy. The XEN gel stent is inserted from the anterior chamber into the subconjunctival space through a limbal approach, using a special insertion device [6]. Before stent insertion, MMC is commonly injected into the subconjunctival space where the bleb is made.

Although ab interno techniques preserve the conjunctiva and Tenon’s capsule relatively well [7], filtration success depends on a well-functioning diffuse bleb, which indicates adequate aqueous flow from the anterior chamber into the subconjunctival space [1]. The natural healing process of inflammation leads to subconjunctival and episcleral fibrosis, which can subsequently lead to bleb failure [3] by limiting outflow (thus increasing IOP) [8]. MMC remains the most frequently used agent due to its reasonable efficacy in preventing fibrosis. An increased therapeutic success rate was reported after XEN implantation by decreasing fibrotic reactions [9]. Subconjunctival injection of 0.01% MMC reduced the rate of needling to 30.7% [2]. In another study, MMC provided near-complete inhibition of fibroblastic proliferation [10]. Therefore, subconjunctival application of MMC is currently considered as a routine procedure during XEN gel stent implantation. Regarding the efficacy of different concentration of MMC during XEN gel stent implantation, a recent study showed two concentrations of MMC 0.01% and 0.02% presented similar effect on IOP lowering, and had no significant differences on the occurrence of adverse events or success rate [11]. Mostly, it is known that subconjunctival MMC injection is performed before XEN implantation [1]. If MMC injection is performed after XEN implantation, MMC may reflux into the anterior chamber and cause toxicity to eyeball [1]. Therefore, to avoid side effect it is recommended to perform MMC injection into the Tenon’s area, at least 8 mm away from the limbus [1].

Despite the use of MMC during the implant procedure, it has been reported that XEN gel stent requires more frequent bleb needling compared to conventional trabeculectomy [3]. Due to such inherent problem, various alternate ways of implanting the XEN gel stent are now being explored, including varying the amount and mode application of MMC [2], and the depth of placement of the implant, as well as preimplantation subconjunctival injection of viscoelastic or air [1], postoperative conjunctival manipulation with blunt instruments, and even “on-table” needling [3]. The XEN air technique allows for more comfortable and reliable subconjunctival placement of the XEN gel stent, via viscoelastic and pneumatic dissection of the subconjunctival space [1]. This provides a stable pocket for XEN stent insertion and minimizes the risk of blockage of the stent by Tenon’s capsule [1].

To our knowledge, this is the first case report of MMC toxicity after subconjunctival OVD injection during XEN gel stent implantation. The present case showed persistent diffuse conjunctival hyperemia with large avascular bleb, corneal stroma haze and mild to moderate degree of ptosis, which may be considered as MMC toxicity. Other previously reported MMC toxicity after glaucoma filtering surgery include bleb leak, blebitis, corneal epithelial toxicity, corneal or scleral melting, and hypotony, which were not observed in the present case. [5, 12, 13] A possible cause of the MMC toxicity after XEN gel stent implantation in the present case may be subconjunctival OVD and MMC injection. As described above, MMC increases the success rate of glaucoma filtration surgery, but may cause toxicity if not properly absorbed such that it remains in the conjunctival tissue. In our case, the absorption of MMC was likely hindered by the OVD, such that it may have accumulated in the subconjunctival space; toxicity occurred due to persistent exposure to the accumulated MMC, leading to post-surgical complications. Therefore, surgeons should be aware of this potential complication and carefully observe patients during the early postoperative period.

In summary, we report a case of MMC toxicity after ab interno XEN gel stent implantation using the XEN air technique. A cautious approach is required during XEN gel stent implantation when performed in conjunction with subconjunctival OVD and MMC, and thorough post-operative follow-up may be necessary to monitor signs of MMC toxicity.

## Data Availability

All data and materials supporting our findings are contained within this manuscript.

## Abbreviations

MMC: Mitomycin C
IOP: Intraocular pressure
OVD: Ophthalmic viscosurgical device
HVF: Humphrey visual field
OCT: Optical coherence tomography
MIGS: Minimally invasive glaucoma surgery
KP: Keratic precipitates
RNFL: Retinal nerve fiber layer
